# Ingestion of *Lactobacillus rhamnosus* modulates chronic stress-induced feather pecking in chickens

**DOI:** 10.1038/s41598-021-96615-x

**Published:** 2021-08-24

**Authors:** Claire Mindus, Nienke van Staaveren, Aadil Bharwani, Dietmar Fuchs, Johanna M. Gostner, Joergen B. Kjaer, Wolfgang Kunze, M. Firoz Mian, Anna K. Shoveller, Paul Forsythe, Alexandra Harlander-Matauschek

**Affiliations:** 1 Department of Animal Biosciences, University of Guelph, 50 Stone Road East, Guelph, ON N1G 2W1 Canada; 2 Michael G. DeGroote School of Medicine, McMaster University, 1280 Main Street West, Hamilton, ON L8S 4K1 Canada; 3 Institute of Biological Chemistry, Biocenter, Center for Chemistry and Biomedicine, Medical University of Innsbruck, Innsbruck, Austria; 4 Institute of Medical Biochemistry, Biocenter, Center for Chemistry and Biomedicine, Medical University of Innsbruck, Innsbruck, Austria; 5 Institute of Animal Welfare and Animal Husbandry, Friedrich-Loeffler-Institut, Celle, Germany; 6 Brain-Body Institute, St. Joseph’s Healthcare, McMaster University, 1280 Main Street West, Hamilton, ON L8S 4K1 Canada; 7 Division of Respirology, Department of Medicine, McMaster University, 50 Charlton Avenue East, Hamilton, ON L8N 4A6 Canada

**Keywords:** Animal behaviour, Animal physiology

## Abstract

Feather pecking (FP) is a stress-induced neuropsychological disorder of birds. Intestinal dysbiosis and inflammation are common traits of these disorders. FP is, therefore, proposed to be a behavioral consequence of dysregulated communication between the gut and the brain. Probiotic bacteria are known to favorably modulate the gut microbiome and hence the neurochemical and immune components of the gut-brain axis. Consequently, probiotic supplementation represents a promising new therapeutic to mitigate widespread FP in domestic chickens. We monitored FP, gut microbiota composition, immune markers, and amino acids related to the production of neurochemicals in chickens supplemented with *Lactobacillus rhamnosus* or a placebo. Data demonstrate that, when stressed, the incidence of FP increased significantly; however, *L. rhamnosus* prevented this increase. *L. rhamnosus* supplementation showed a strong immunological effect by increasing the regulatory T cell population of the spleen and the cecal tonsils, in addition to limiting cecal microbiota dysbiosis. Despite minimal changes in aromatic amino acid levels, data suggest that catecholaminergic circuits may be an interesting target for further studies. Overall, our findings provide the first data supporting the use of a single-strain probiotic to reduce stress-induced FP in chickens and promise to improve domestic birds' welfare.

## Introduction

The mammalian stress response initiates a cascade of physiological and behavioral changes through the neuroendocrine, metabolic, immune, and autonomic nervous systems^1^. When stressors are severe, chronic, multiple, and/or unpredictable, these adaptation processes may fail to return the organism to homeostasis^2^. This failure can lead to chronic disease, which is accompanied by increased hypothalamic–pituitary–adrenal (HPA) axis activity^3^, severe decrease in number and function of lymphocytes T subset profiles^4^, changes to concentrations of immune activation markers^5,6^, and inflammation-induced changes in catabolism of aromatic amino acids, such as tryptophan (TRP), phenylalanine (PHE) and tyrosine (TYR)^7^.

Disturbances in monoaminergic neurotransmitters systems that regulate behavior^8^, in their aromatic amino acids precursors’ metabolism as well as in the HPA-axis such as observed in psychiatric disorders^9–11^, are concomitant with altered functioning and metabolism of lymphocytes^12^. Lymphocytes are cells of the immune system divided into two major lineages which include the T (thymus-derived) and B (bone-marrow-derived, or bursa of Fabricius–derived in avian species) cells^13^. Subsets of T lymphocytes (distinguished by surface-markers) are responsible for cell-mediated immunity and cytotoxicity but also regulate the inflammatory response^14^ and, despite some differences^15^, can be found both in mammals and in chickens^16^.

In mammals, TRP is largely catabolized into kynurenine (KYN) via multiple enzymes, namely, indoleamine 2,3-dioxygenase 1 and 2 (IDO-1, IDO-2), and tryptophan 2,3-dioxygenase (TDO), with a small percentage being converted into serotonin. PHE is metabolized to TYR to produce the precursor of the catecholamine neurotransmitters dopamine, adrenalin, and noradrenalin. Stress-induced immune activation can dampen the catabolism of these aromatic amino acids into neurochemicals^7,17^. In turn, in mammals, a pro-inflammatory state decreases serotonin and catecholamine production^18–21^. Actually, one of the most potent inducers of IDO is the cytokine interferon-γ, produced by T lymphocytes^22^. Similar to mammals, stress can suppress the chicken immune system^23^. However, there is some indication that the avian amino acid metabolism may be less intimately linked to stress and the immune response compared to mammals by virtue of enzymatic differences in the catabolic pathways^24^.

Recent research suggests that stress-induced activation of the neuroendocrine system affects gastrointestinal tract function^25^, including gastrointestinal motility^26^ and gut microbiota composition^27–30^. For instance, exposure to stress reduces beneficial microbes, such as *Lactobacillus* species^31–33^. The resulting dysbiosis may disrupt the epithelial barrier, thereby increasing susceptibility to enteric infection and inflammation^34^. This “leaky gut” phenomenon allows bacteria to translocate across the intestinal mucosa and activates a mucosal immune response increasing the production of pro-inflammatory cytokines^10,18^.

The majority of commercial laying hens hatch and live in standard housing, which tends to be physically, socially, nutritionally, and sensorially restricted environments. These environments can be powerful social and environmental chronic stressors that could induce high rates of severe feather pecking (FP)^35^. While the gentle form of FP in laying hens targets the tips and edges of feathers without causing damages and is suggested to be similar to social exploration or allo-preening^35^, the severe form of FP is a behavior, whereby individuals grasp, pull and occasionally ingest feathers of their conspecifics^36^. FP can cause feather loss and skin damage, and in some cases, this can escalate to severe injuries and cannibalism^35^. The prevalence of severe FP is reported to range from 15 to 95% on laying hen commercial farms, making it a major animal welfare concern^37,38^.

Similarly to human psychiatric disorders, FP is associated with a range of comorbidities, such as higher HPA-axis reactivity^39,40^, alterations in the monoaminergic neurotransmitter system and their precursors, TRP, PHE, and TYR^24,41–44^, and despite no obvious difference in relative abundance T lymphocytes subsets, a more sensitive immune system^45,46^. Of the multiple comorbidities accompanying FP, the role of the gut-brain axis in the development of FP has received increased attention in recent years. For example, Birkl et al.^47^ and van der Eijk et al.^48^ demonstrated that the cecal droppings of FP birds had a higher abundance of *Clostridiales* and a lower abundance of *Lactobacillus* compared to non-FP birds. Additionally, FP and non-FP birds are reported to harbor distinct gut microbiota and short-chain fatty acid profiles^49^. Whether such differences contribute to FP symptoms or whether they are the underlying cause of FP is still unknown. Despite the vast complexity of the gut microbiota, research in human and murine models show that supplementation with a single or multiple bacterial strain(s) can protect against stress-induced behavior by shifting the levels of monoamine neurotransmitter precursors, by altering the immune response such as increasing T regulatory cells (a lymphocyte subset), and by reversing gut dysbiosis^50–52^.

In this study, we investigated the ability of oral supplementation with a single bacterium to modulate FP behavior, immune biomarkers and T cell phenotypes, as well as cecal microbial composition, the HPA axis and aromatic amino acid metabolism in birds selected for FP and exposed to chronic, repeated, unpredictable stress. To this end, we used *Lactobacillus rhamnosus* JB-1 for the oral supplement as the *Lactobacillus* genus is underrepresented in the cecal droppings of feather peckers^47,48^. Furthermore, *L. rhamnosus* has been demonstrated to attenuate behavioral deficits induced by chronic social stress in mice^52^.

## Results

### *Lactobacillus rhamnosus* supplementation modulates stress-induced pecking behavior and feather cover

**Figure 1:**
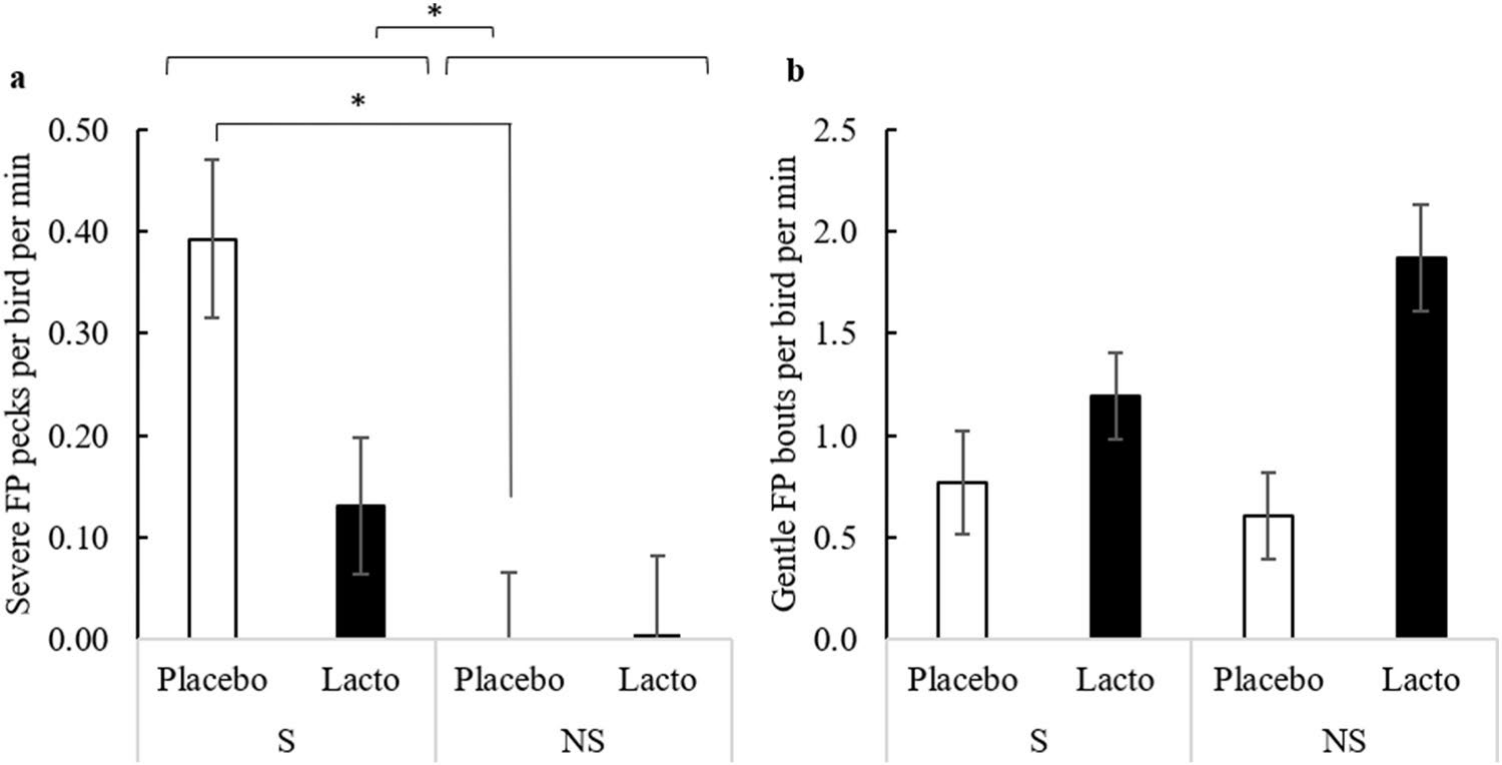
Expression of severe and gentle feather pecking behavior. Frequency of severe (**a**) and gentle (**b**) feather pecks per minute (Least Squares Means ± Standard Error) between weeks 24 and 26 based on supplementation (Lacto: *L. rhamnosus*, Placebo: Placebo supplementation) and stress (S: stressed, NS: non-stressed) treatment (n: S-Placebo = 22, NS-Placebo = 22, S-Lacto = 20, NS-Lacto = 22). Asterisks (*) indicate statistically significant differences (P < 0.05).

The sequence of stressors in the present study triggered severe feather pecking (FP) in birds supplemented with *L. rhamnosus* (Lacto) or with drinking water as a control (Placebo) (F_1,6_ = 9.56, P = 0.021, Fig. 1a). As expected, this was accompanied by a significant increase in the damage to the feather cover (OR = 4.10, 95% CI 1.14–14.78, F_1,82_ = 4.78, P = 0.032).

Interestingly, the Lacto treatment significantly attenuated severe FP behavior in the presence of stress by 2.5-fold relative to birds receiving the Placebo (Fig. 1a). Indeed, the incidence of severe FP (P = 0.664) and the feather cover score (OR = 1.15, 95% CI 0.31–4.25, P = 0.996) in stressed, Lacto birds was not significantly different from that of non-stressed Lacto birds. In contrast, stressed, Placebo supplemented birds performed a significantly higher number of severe FP events (P = 0.032, Fig. 1a) and displayed a tendency to have a more deteriorated feather cover than their non-stressed counterparts (OR = 14.54, 95% CI 1.59–132.70, P = 0.084). It is also noteworthy that Lacto birds performed gentle FP more frequently than Placebo birds (F_1,6_ = 11.94, P = 0.014; Fig. 1b), regardless of the stress treatment.

### *Lactobacillus rhamnosus* treatment prevents stress-induced alterations to the cecal microbiota

**Figure 2:**
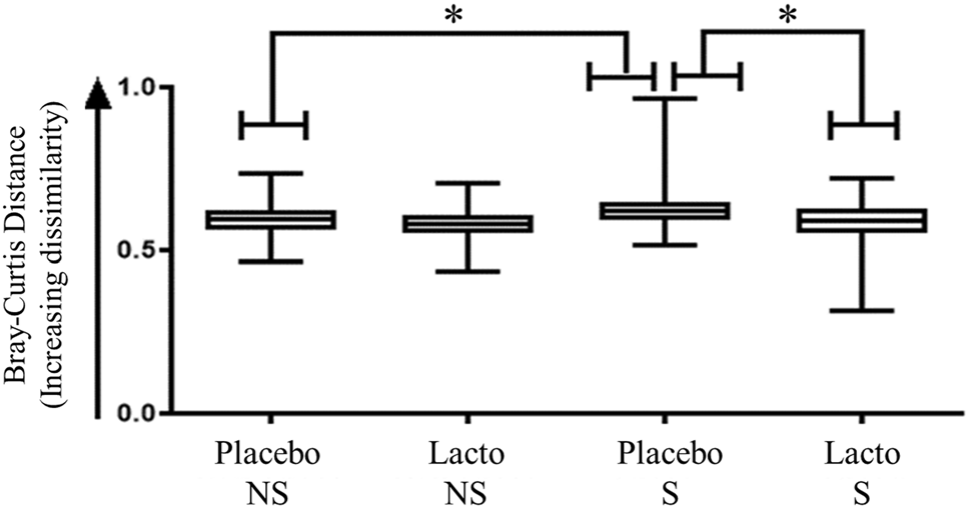
Alpha diversity of microbial communities of cecal samples from laying hens. Alpha diversity of microbial communities of cecal samples measured using the Shannon index from laying hens at 27 weeks of age based on supplementation (Lacto: *L. rhamnosus*, Placebo: Placebo supplementation) and stress (S: stressed, NS: non-stressed) treatment (n: S-Placebo = 22, NS-Placebo = 22, S-Lacto = 18, NS-Lacto = 22).

The species richness of the microbial communities (i.e., alpha diversity) was measured using the Shannon’s diversity index. We found that alpha diversity was similar between the test groups and was not influenced by either Lacto supplementation (F_1,80_ = 0.380, P = 0.539) or stress (F_1,80_ = 0.425, P = 0.516; Fig. 2). Changes in relative abundance of individual operational taxonomic units are shown in Supplementary Figure S1.

**Figure 3:**
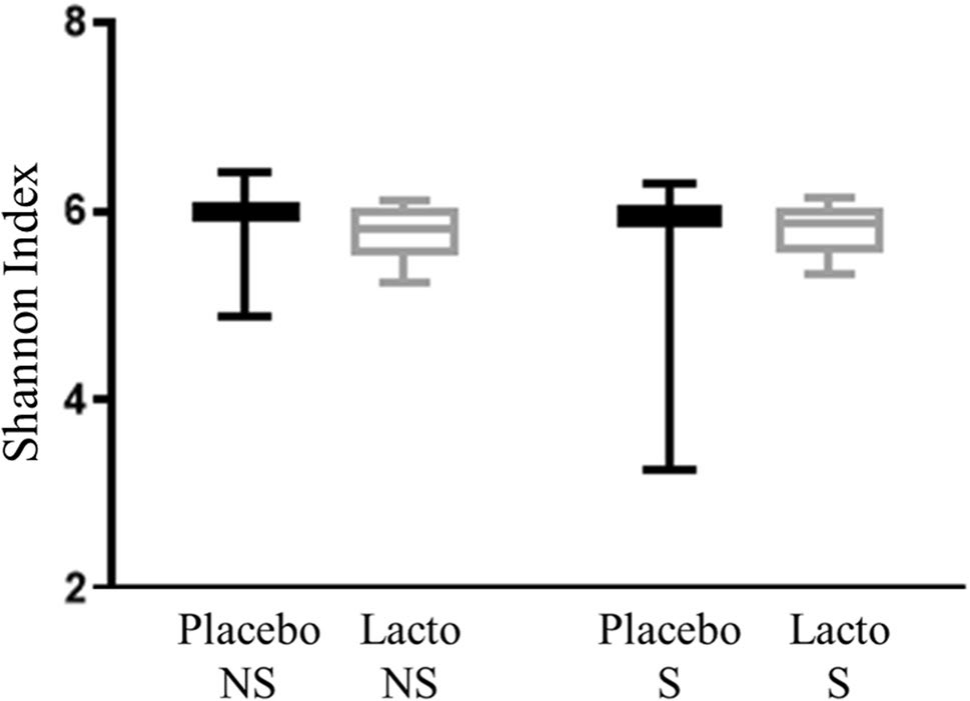
Beta diversity of microbial community diversity of cecal samples from laying hens. Beta diversity analysis as shown by Bray–Curtis distance calculations. The dissimilarity values for each experimental group based on supplementation (Lacto: *L. rhamnosus*, Placebo: Placebo supplementation) and stress (S: stressed, NS: non-stressed) treatment compare week 18 (baseline) and week 27 (n: S-Placebo = 22, NS-Placebo = 22, S-Lacto = 18, NS-Lacto = 22). Asterisks (*) indicate statistically significant differences (P < 0.05).

Beta diversity accounts for differences in the bacterial community composition. In contrast to alpha diversity, beta diversity measured using the Bray–Curtis dissimilarity, was significantly altered by chronic, unpredictable stressors in the Placebo birds (median: NS-Placebo: 0.594, S-Placebo: 0.619; P < 0.05; Fig. 3). Stressed Placebo birds also had a higher dissimilarity value relative to the baseline (18 weeks of age [woa]) than stressed Lacto supplemented birds (median: S-Placebo: 0.619, S-Lacto: 0.590; P < 0.05; Fig. 3).

### *Lactobacillus rhamnosus* supplementation regulates chronic stress-induced alterations of T-regulatory cells population in the spleen and cecal tonsils

**Figure 4:**
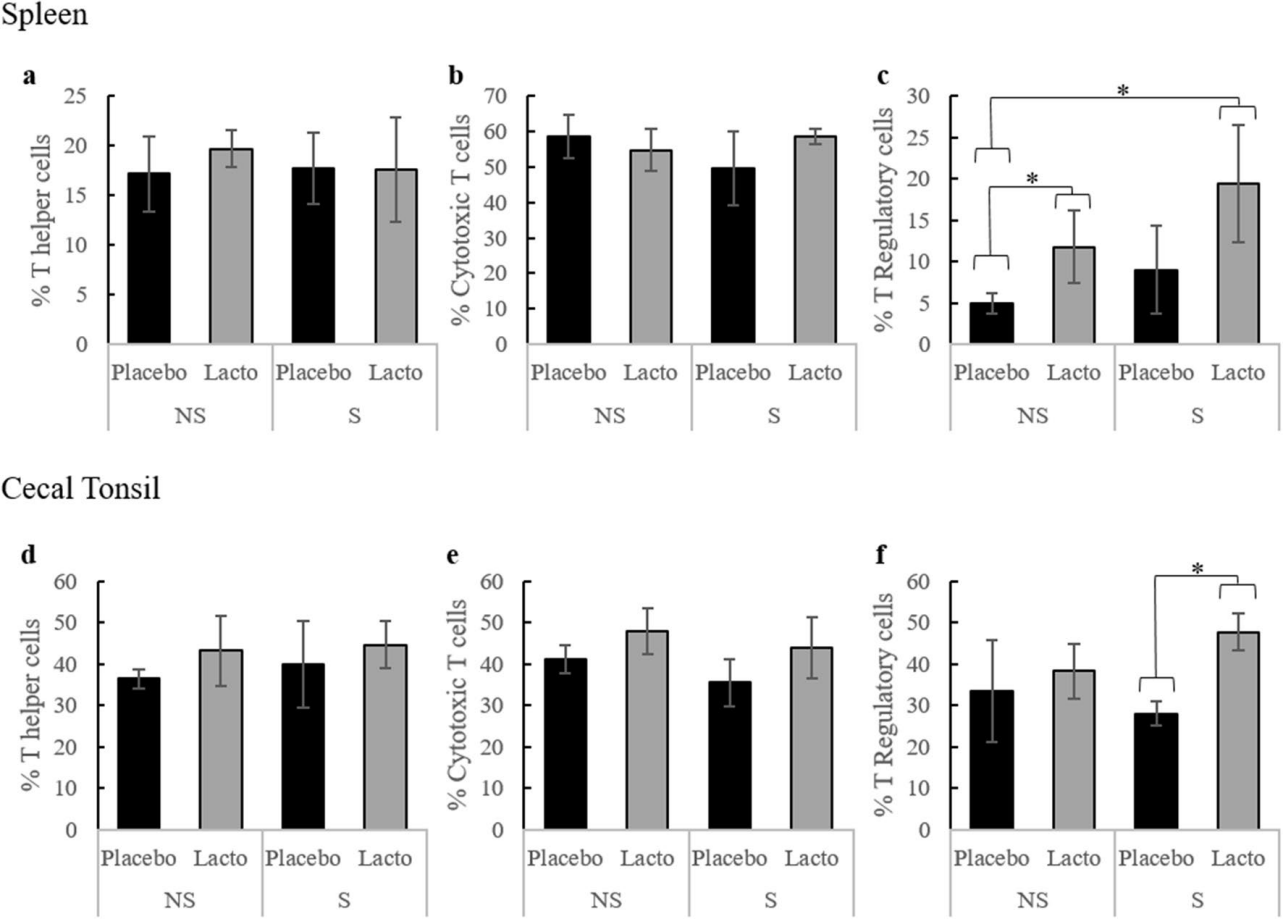
T cell sub-populations in the spleen and cecal tonsils. T cell sub-populations in the spleen (**a**–**c**) and cecal tonsils (**d**–**f**) after three weeks of *L. rhamnosus* (Lacto) or placebo (Placebo) supplementation and stress treatment (S: stressed, NS: non-stressed). Sub-populations were identified using the following combinations of cell surface markers: T helper cells = CD3 ^+^ CD4 ^+^ ; cytotoxic T cells = CD3 ^+^ CD8 ^+^ ; T regulatory cells = CD4 ^+^ CD25 ^+^ (n: S-Placebo = 4, NS-Placebo = 4, S-Lacto = 4, NS-Lacto = 4). Asterisks (*) indicate statistically significant differences (P < 0.05).

The common T cell marker, CD3 was used in combination with other cell surface markers to identify changes to T cell sub-populations in response to *L. rhamnosus* supplementation and the sequence of stressors introduced in this experiment. An example of the gating strategy is shown in Supplementary Figure S2. According to flow cytometry analysis, neither Lacto supplementation nor stress impacted the proportion of T helper cells (CD3^+^CD4^+^) and cytotoxic T cells (CD3^+^CD8^+^) in the spleen (Fig. 4a,b) or in the cecal tonsils (Fig. 4d,e).

In contrast, we report a significant increase of the proportion of regulatory T cells (Treg; CD4^+^CD25^+^) in the cecal tonsils (P < 0.001, Fig. 4f) and a tendency of increase in the spleen (P = 0.088, Fig. 4c) in stressed Lacto birds compared to stressed Placebo birds. Indeed, Treg cells represented 47.6% of the cecal tonsil T cell population in the former group, while this proportion was merely 28% in the latter (F_3,3_ = 2.18, P < 0.001). In non-stressed birds, splenic Treg cells were also significantly more abundant in Lacto birds compared to Placebo birds (11.75% vs. 5.0% F_3,3_ = 12.79, P = 0.042). This difference in the spleen was further increased when Lacto birds were stressed (F_3,3_ = 33.61, P = 0.014). In the absence of stress, the proportion of Treg cells in the cecal tonsils were similar between groups.

### Severe FP phenotype is associated with elevated PHE levels

Stressors, *L. rhamnosus* supplementation and their interaction did not significantly change peripheral plasma levels of TRP, PHE, TYR, KYN, and their relevant ratios. Furthermore, the TRP:(PHE + TYR) ratio, as well as the nitrite, neopterin and corticosterone (CORT) concentrations were similar between groups (Table 1).

**Table 1:** Least Squares Means (± Standard Error) of amino acid, immune biomarker and corticosterone concentrations in laying hens (n: S-Placebo = 22, NS-Placebo = 22, S-Lacto = 20, NS-Lacto = 22) after 8 weeks of treatment based on supplementation (Lacto: *L. rhamnosus*, Placebo: Placebo supplementation) and stress (stressed, non-stressed).

	Lacto (n = 42)		Placebo (n = 44)		P-value
	Stressed (n = 20)	Non-Stressed (n = 22)	Stressed (n = 22)	Non-Stressed (n = 22)	
Tryptophan (TRP) (µmol/L)	73 ± 2.3	71 ± 2.1	72 ± 2.1	75 ± 2.1	0.2914
Tyrosine (TYR) (µmol/L)	93 ± 4.6	94 ± 4.6	93 ± 4.3	96 ± 4.6	0.7874
Phenylalanine (PHE) (µmol/L)	90 ± 3.7	90 ± 3.6	91 ± 3.3	92 ± 3.6	0.8802
TRP:(PHE + TYR)	0.40 ± 0.019	0.40 ± 0.019	0.40 ± 0.017	0.41 ± 0.019	0.9692
KYN:TRP (µmol/mmol)	3.0 ± 0.32	3.9 ± 0.60	3.2 ± 0.41	2.8 ± 0.30	0.1119
PHE:TYR (µmol/µmol)	1.00 ± 0.032	0.98 ± 0.033	0.98 ± 0.030	0.97 ± 0.032	0.8376
Kynurenine (KYN) (µmol/L)	0.22 ± 0.021	0.29 ± 0.040	0.21 ± 0.024	0.22 ± 0.021	0.3222
Nitrite (µmol/L)	53 ± 9.8	37 ± 6.5	50 ± 10.8	49 ± 8.7	0.3843
Neopterin (nmol/L)	2.6 ± 0.11	2.55 ± 0.089	2.63 ± 0.095	2.60 ± 0.087	0.7889
Corticosterone (pg/mL)	2093 ± 538.1	1644 ± 423.6	1451 ± 359.0	1270 ± 308.9	0.8277

No statistically significant difference was found due to the stress treatment, *L. rhamnosus* supplementation or their interaction (P-value shown refers to the interaction).

However, birds exhibiting a severe FP phenotype, characterized as individuals that displayed at least once severe FP bout between 24–26 woa, had higher peripheral plasma PHE levels (F_1,77_ = 7.88, P = 0.006) and a tendency for higher TYR levels (F_1,77_ = 3.84, P = 0.054) compared to birds classified as non-feather peckers (Table 2).

**Table 2:** Least squares means (± Standard Error) of the amino acid, immune biomarker and corticosterone concentrations in laying hens according to their feather pecking phenotype.

	Feather pecker (n = 30)	Non-feather pecker (n = 56)	P-value
Tryptophan (TRP) (µmol/L)	74 ± 1.8	72 ± 1.3	0.2444
Tyrosine (TYR) (µmol/L)	100 ± 3.6	91 ± 2.7	0.0537
Phenylalanine (PHE) (µmol/L)	97 ± 2.8	87 ± 2.1	0.0063
TRP:(PHE + TYR)	0.39 ± 0.015	0.41 ± 0.011	0.2723
KYN:TRP (µmol/mmol)	3.1 ± 0.26	3.1 ± 0.21	0.8416
PHE:TYR (µmol/µmol)	0.99 ± 0.026	0.98 ± 0.019	0.7619
Kynurenine (KYN) (µmol/L)	0.24 ± 0.020	0.23 ± 0.015	0.7218
Nitrite (µmol/L)	50 ± 7.5	45 ± 4.6	0.6043
Neopterin (nmol/L)	2.53 ± 0.077	2.68 ± 0.059	0.1370
Corticosterone (pg/mL)	2180 ± 528.2	2278 ± 398.3	0.8842

A feather pecker was characterized as a bird that displayed severe feather-pecking behavior at least once between 24–26 weeks of age.

## Discussion

**Figure 5:**
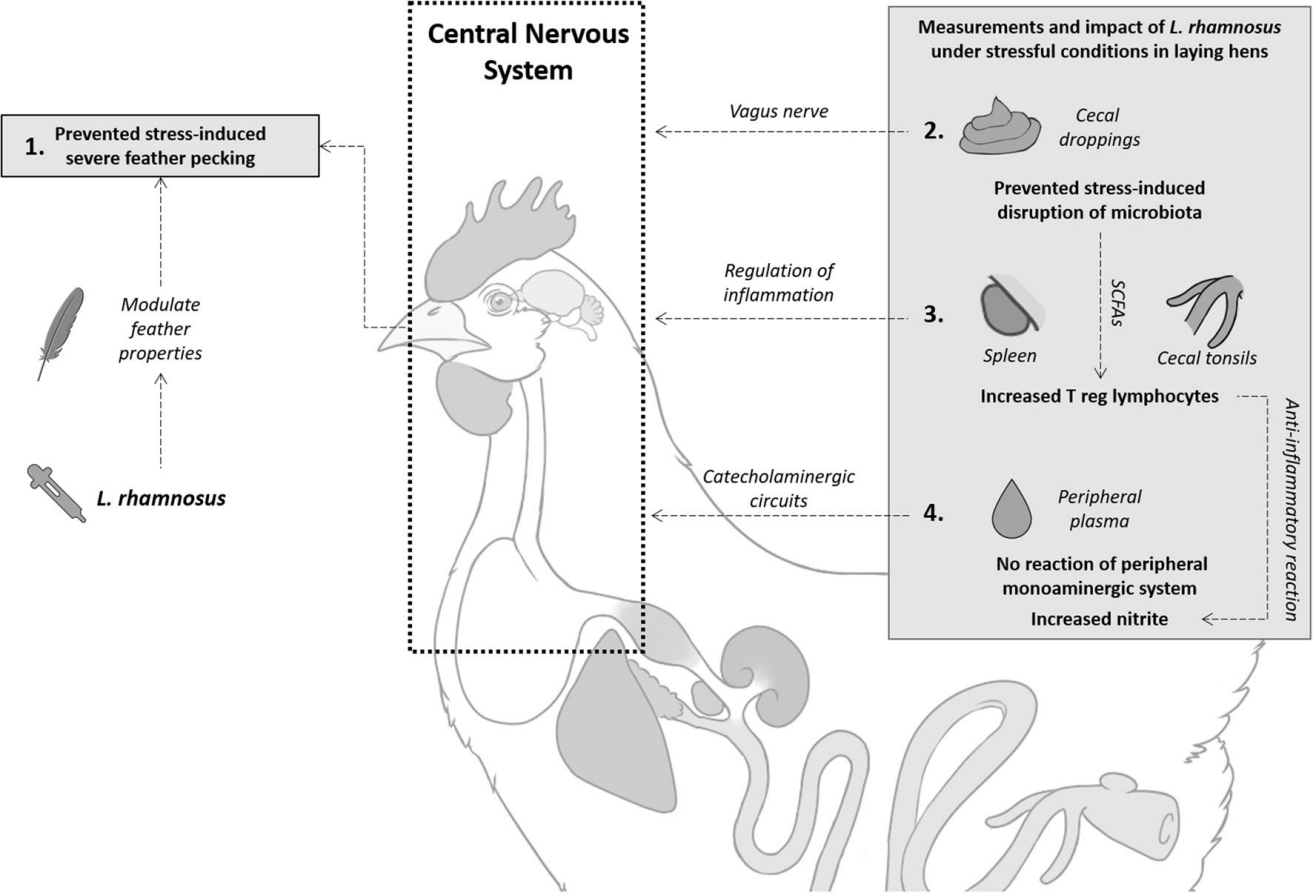
Effect of *Lactobacillus rhamnosus* on feather pecking behavior, related physiological pathways, and hypothetical mode of action of the bacteria in chronically stressed laying hens. *L. rhamnosus* prevented a stress-induced increase of severe feather pecking in chickens (1); potentially the *L. rhamnosus* modulates the sensory properties (e.g., taste, smell) of feathers via transfer of oral microbiota to the feather cover. However, L*. rhamnosus* supplementation impacts more physiological systems as shown by limiting stress-induced cecal microbiota dysbiosis (2). This may impact the enteric nervous system, notably the nervus vagus, which could induce changes in the cerebral GABAergic activity. Changes in the birds’ immune system were found that indicate a role for the regulation of inflammation as shown by an increase in regulatory T (Treg) cell populations in the spleen and cecal tonsils (3). *L. rhamnosus* could potentially produce short chain fatty acids (SCFAs) that are known to trigger an increase in Treg in other animals. This increase in Tregs could be a counter reaction to stressed-induced inflammation as shown by elevated nitrite concentration in the peripheral plasma (4). While alterations in the monoaminergic system are implicated in feather pecking behavior, we found limited changes in the peripheral monoaminergic system (4), however, catecholaminergic circuits could still play a role. Changes in these different physiological pathways can influence the central nervous system, and ultimately behavior, such as feather pecking in laying hens. Experimental findings are numbered (1–4) and hypothetical modes of actions of L. rhamnosus are represented with dashed arrows.

Recent advances in feather pecking (FP) research in laying hens explore the role of bidirectional communication between the gut and the brain and have suggested that severe FP may be linked to neurobiological dysfunction^35^. Fundamental work has shown that the gut microbiota and the microbiota-derived metabolites are distinct in feather peckers versus non-peckers, whereby the former has a significantly lower abundance of *Lactobacillus* bacteria in the ceca^47–49^. In murine models, specific *Lactobacillus* gut bacteria can modulate central nervous system-driven behaviors^50,52^, alter the monoaminergic system^53^ and their amino acid precursors^54,55^, impact the hypothalamic–pituitary–adrenal (HPA) axis^50^, and influence the immune system^52,56–58^. Interestingly, these same pathways are associated with severe FP in laying hens^24,41,43,45,46,59–61^. Nevertheless, the ability of a single bacterial strain to prevent or reduce FP and/or impact the associated physiological systems has not yet been investigated. Therefore, the aim of the present study was to determine whether oral supplementation with a single *Lactobacillus rhamnosus* strain could modulate stress-induced FP behavior and associated physiological parameters in laying hens that are genetically selected for high FP activity. To this end, we report the effect of stress and *L. rhamnosus* supplementation on FP, T cell phenotypes, the cecal microbiota, corticosterone levels, and the metabolism of the aromatic amino acids tryptophan (TRP), phenylalanine (PHE), and tyrosine (TYR) (Fig. 5).

The sequences of stressors administered to the experimental subjects of this study was designed to mimic the chronic, unpredictable, social and non-social environments that laying hens encounter in commercial settings. The FP data (Fig. 1a) mirror the literature, showing that severe FP increased in response to stress ^24^. A critical finding of this study is that an oral *L. rhamnosus* supplement (Lacto group) prevented the stress-induced increase of severe FP behavior (Fig. 1a) and concomitantly improved the feather cover, a reliable indicator of severe FP^62,63^. Our results suggest that a single bacterial strain is thus protective against social behavioral deficits triggered by chronic, unpredictable environments in laying hens consistent with data from studies using murine models^50,52^.

In addition to its ability to prevent severe FP, the beneficial effect of an *L. rhamnosus* supplement is further evidenced by the increase of gentle FP in Lacto birds (Fig. 1b). Gentle FP has been suggested to be a form of explorative, pro-social pecking that plays an important role in the building and maintenance of positive, social relationships among chicks^35^. Given the divergent motivations behind gentle and severe FP, it is critical that studies investigating FP differentiate these two forms^35^. In mice, *L. rhamnosus* likewise mediates pro-social behavior which is accompanied by changes in cerebral GABAergic activity via the vagus nerve^50^. A recent study showed that *L. rhamnosus* interacts with the enteric nervous system (ENS) in laying hens^64^. Considering that the vagus nerve connects the ENS with the central nervous system, the ENS may indeed be an important component of the pathway through which *L. rhamnosus* promotes sociable, gentle FP behavior in chickens. Further research is required to test this assumption.

While oral supplementation focuses on the microbiota-gut-brain axis, it is important to acknowledge the potential impact of this treatment on the oral microbiota and its possible consequences on FP behavior. Indeed, the transfer of the oral microbiota to the feather cover via preening or pecking may change the olfactory features or taste of feathers. Enteric and environmental bacteria influence olfaction in mice^65^, alter body odor in birds^66^ and are responsible for recognition of species^67^. Thus, it is important to acknowledge that, in the present study, oral supplementation of *L. rhamnosus* may have resulted in the transfer of the bacteria to the feather cover via preening or pecking leading to a change in the olfactory features or taste of feathers^36^. This is turn, may have promoted gentle FP and dissuaded birds from performing severe FP. While the theory needs to be tested experimentally, this association may identify the feather cover or skin of the birds as a significant contributor to social behavior.

In addition to reducing severe FP and improving feather cover, our data show that the stress treatment significantly altered the microbiota profile of the cecal droppings. The stressed Placebo hens had a higher increase in dissimilarity from 18 to 27 weeks compared to the non-stressed Placebo hens (Fig. 3). However, the stress treatment did not alter the changes in the microbiota profile between 18 and 27 weeks in Lacto hens (Fig. 3). Additionally, the stressed-Lacto birds displayed significantly lower dissimilarity in their cecal microbial community diversity from week 18 to 27 than the stressed-Placebo. There were also OTU level differences between stressed Placebo hens and Lacto hens (Figure S1). This suggests that *L. rhamnosus* supplementation is preventing stress-induced disruption of cecal bacterial community structure in laying hens. However, regardless of the bird’s supplementation and stress treatments, no significant correlations were found between the Shannon Index values and the bird’s gentle and severe pecking phenotype (data not shown). It should be noted that we only assessed the effect of Lacto treatment under stressful condition on microbiome composition. Indeed, previous studies have demonstrated that *L. rhamnosus* shows behavioral effects under challenging, i.e., stressful situations and does so without altering microbiome composition^52^. Furthermore, it has been observed that the *L. rhamnosus* can induce changes within minutes on the gut motility ex-vivo in mice^68^ and in laying hens^64^, further suggesting that the effect of *Lactobacillus* may be mediated through other avenues than microbiota composition changes. Thus, the effects of supplementation alone on microbiome would provide no mechanistic insight. The actual mode of action of L. *rhamnosus* is yet to be shown experimentally.

The immunomodulatory capacities of *L. rhamnosus* are well recognized in mammals^57,69,70^. We presently report that the *L. rhamnosus* supplement also impacts immune measurements in birds. In particular, *L. rhamnosus* supplementation significantly increased the regulatory T (Treg) cell population in the cecal tonsils and the spleen in response to stress (Fig. 4). Regulatory T cells are suppressor T cells crucial for regulating the inflammatory response and preventing autoimmunity^14^. Thus, we conclude that the bacterium was able to influence the local immune system of the hens similar to previous observations in mice^57,69^. Chicken cecal tonsil cells respond more rapidly than spleen cells to the bacterial stimuli, which could explain differences between these tissue sites^71^. Short-chain fatty acids (SCFAs) produced by commensal organisms are known to trigger the increase of Treg in mammals and chicken cecal tonsils^72–74^. The ability of *L. rhamnosus* to produce SCFAs that mediate immune activation is yet to be investigated, and further research should consider testing for SCFAs to help identify the modes of action of the bacteria.

Nitric oxide (NO) is produced as part of the innate, pro-inflammatory immune response when nitric oxide synthase production is stimulated by IFN-γ. Kujundžić and Lowenthal^75^ demonstrated that the expression of inducible nitric oxide synthase (iNOS) and NO formation can be efficiently stimulated by chicken IFN-γ in the chicken macrophage cell line HD11, reaching nitrite concentration of approximately 40 µmol/L after 24 h of treatment. Since NO is rapidly converted to the more stable metabolite nitrite, the latter is used as a surrogate marker of NO^76^. We report no statistically significant differences in the nitrite levels between treatment groups. However, under non-stressed conditions, the Lacto birds had a lower plasma nitrite concentration compared to the Placebo group. Moreover, in parallel to increasing Treg cells, stress caused an increase in nitrite levels in Lacto supplemented birds. Taken together, this set of data suggests that the baseline inflammation status is lower in Lacto-supplemented birds. The overall increase of the Treg population in stressed Lacto birds may be an attempt to counteract the induced immune activation^77^. Thus, the pro-inflammatory response leading to more nitrite may be balanced out by the anti-inflammatory effect of Treg cells on the long term.

Corticosterone (CORT) is a surrogate measure of the HPA axis activation and is used as an indicator of acute stress in birds^42,78^. Surprisingly, we observed no increase in the plasma CORT concentration under stress. Nevertheless, it is noteworthy that CORT levels recorded in this study were low compared to a recent study using the same genetic line^45^ and similar to those in resting birds^40^. We further report that CORT measurements between peckers and non-peckers were similar, confirming previous observations^61^. Despite being an indicator of acute stress, chronic stress is known to downregulate CORT production through a negative feedback loop^79^. Therefore, it is possible that the sequence of stressors used in the study dampened CORT levels over time, as this stress regimen was intended to represent chronic stress. In addition, it is important to acknowledge that only two timepoints were used to measure plasma CORT levels, which may not have adequately represented changes in CORT concentrations under chronic stress conditions.

Neither the ingestion of *L. rhamnosus* nor the chronic use of stressors significantly altered TRP, PHE, and TYR levels, their ratios, or the downstream metabolite, KYN. In humans, an elevated KYN:TRP ratio is an indicator of TRP degradation by the IDO-1 enzyme activated during acute innate and adaptive immune responses^7,18–20^, linking immune activation to the development of neuropsychiatric symptoms^7^. Actually, one of the most potent inducers of IDO is IFN-γ, produced by T cells^22^, while certain IDO-expressing cells inhibit T cell activation^80,81^. In parallel, pro-inflammatory cascades are also associated with increased phenylalanine hydroxylase (PAH) activity^21^, which metabolizes PHE to TYR and is reflected by the PHE/TYR ratio. In chronic episodes of immune activation, PHE and PHE/TYR ratio are elevated at the expense of dopamine^82^, a neurotransmitter which has actions on both the nervous and the immune systems^83^ and is involved in gut-brain signaling^84^. Further down the PHE-TYR-Dopamine pathway, norepinephrine differentially regulates naive and effector CD8 ^+^ T cell activity^85^ and decreases functionality and proliferation of CD8 ^+^ T cell^86^. Thus, in mammals, aromatic amino acid metabolism and the monoaminergic neurotransmitter systems are associated and connected to the host peripheral immune system responsiveness. The divergence from the mammal system is likely due to evolutionary differences in laying hen’s amino acid metabolism, notably the lack of IDO-1^87,88^. IDO-2, the isoenzyme, has a relatively low affinity for TRP and a low enzymatic efficiency because of its very low catalytic velocity^87^. So far, no direct in vivo evidence suggests that the induction of aromatic amino acid metabolism in laying hens is mediated via immunological cascades. Interestingly, though Kujundžić and Lowenthal^75^ reported the upregulation of TRP degrading enzymatic activity in IFN-γ stimulated lysates of the chicken macrophage cell line HD11 that is able to produce KYN. Nevertheless, the KYN concentrations of these lysates decreased over a period of 24 h after treatment. It is possible that the activity of other downstream enzymes in the KYN breakdown pathway may be responsible for the consumption of KYN. Therefore, it remains unclear whether cytokines can stimulate TRP catabolism in chicken macrophages.

As this study focuses on laying hens and attempts to mimic commercial settings, it is important to emphasize that data collection coincided with the onset of egg-laying. The nutritional requirements of hens undergo a significant shift during this period, and resources are preferentially channeled towards protein synthesis for egg formation, while requirements for growth are minimal^89^. Additionally, the pullets bred for high FP behavior may not have the same requirement as commercial birds. Thus, we also hypothesize that the respective amino acids concentrations in the commercial diet we used may not have been sufficient for the young laying hens in this study. Indeed, we observed a 14% decrease in plasma TRP and TYR levels between 18 and 26 weeks of age. It is, therefore, possible that the physiological stress encountered by hens during this transitional period may have masked the true changes to plasma amino acid concentrations in response to stress exposure. Finally, there is also evidence suggesting that plasma amino acid levels vary depending on the timing of blood extraction and the amount of feeding. As such, one limitation of this study is that fasting levels of TRP, PHE, and TYR were not recorded.

Yet another important, social context that must be considered, is that the birds in the present study were familiarized with human-interactions which may have attenuated the impact of stressors^40^. Furthermore, social disruptions happened concurrently with the replacement of the bedding material. Fresh bedding material is an environmental enrichment, which, in turn, may have countered the intended stress of the social disruption^90^.

We found that birds that exhibited a severe FP phenotype had significantly higher PHE and a tendency for higher TYR levels than non-peckers (Table 2). However, we report no difference in PHE:TYR ratio between peckers and non-peckers, suggesting that the relative change in PHE and TYR was equal. PHE and TYR can cross the blood–brain barrier. In mammals, the plasma levels are good indicators of PHE and TYR brain levels^91,92^. Peckers may have higher central levels of PHE and TYR producing higher levels of catecholamines, which may indicate a more sensitive and hyperactive response to stress. Thus, our results are in line with the higher dopaminergic activity found in chickens exhibiting severe FP and injurious pecking. This work reinforces the theory that the dopamine pathway plays a role in stress regulation in chickens^24,41,93,94^. However, chickens have different metabolic properties and requirements for amino acids compared with mammals. These results should be considered with caution as the correlation between central and peripheral levels in poultry species is yet to be tested.

To the authors’ knowledge, we show for the first time that administration of *L. rhamnosus* in laying hens can alter brain and immune function under stress, resulting in reduced severe FP and improved feather cover in birds. The gut microbiota was also significantly more resistant to stress-induced changes when birds received the Lacto supplement (Fig. 3). Furthermore, the data presented herein shed light on the potential associations between the immune system, the gut microbiome, and neuro-chemical pathways that contribute to FP.

In conclusion, our data suggest that single-microbe supplementation can ameliorate severe FP in laying hens. Further research should elucidate whether *L. rhamnosus* drives the changes to FP behavior (i.e., direct effect), or whether the bacterium modulates the diversity and/or function of other microbial communities, metabolites, and the immune system, thereby triggering other behavior that result in the observed FP shifts (i.e., indirect effect). Additionally, results would need to be replicated in other genetic lines, as well as commercial lines. The feasibility of supplementation under commercial conditions should also be investigated. Our findings identify potential biological mechanisms behind the therapeutic effects of probiotics which paves the way for individualized, microbial interventions in millions of domestic birds with a history of FP.

## Methods

### Ethical statement

The experiment was approved by the Animal Care Committee at the University of Guelph (Animal Utilization Protocol #3206). The study was carried out in accordance with relevant guidelines and regulations as well as the ARRIVE guidelines^95^.

### Animals and housing conditions

Non-beak-trimmed White Leghorn laying hens, originating from a selection experiment in which birds were divergently selected for high (HFP) and low (LFP) feather pecking behavior^96^, were used in this study. A total of 86 HFP birds were wing tagged at hatch and housed in 4 floor pens (22 ± 5 birds) until 14 weeks of age (woa) under conventional management conditions at the Research Station of the University of Guelph, Guelph, Ontario, Canada.

At 14 woa, birds were randomly allocated to 12 identical floor pens (7 ± 1 birds). Each pen (1.6 m2) was littered with wood shavings as bedding material and equipped with one round metal feeder (43 Ø cm), a 4 nipple drinker, two nest boxes, 1 platform (125 × 31 cm, 90 cm above the ground) and two elevated perches (15 cm of perch/hen; 55 and 120 cm above the ground). One camera (Samsung SNO-5080R, IR, Samsung Techwin CO., Gyeongi-do Korea) was ceiling-mounted above each pen to allow for a full view of the pen. Auditory contact and smell were allowed between pens, but visual contact was prevented by opaque PVC boards. Light was provided at 20 lx from 05:00 until 19:00. The average daily temperature was 20 ± 0.5 °C. Birds had ad libitum access to well water and commercial layer feed.

### *Lactobacillus rhamnosus* supplementation and chronic, unpredictable stress treatments

**Figure 6:**
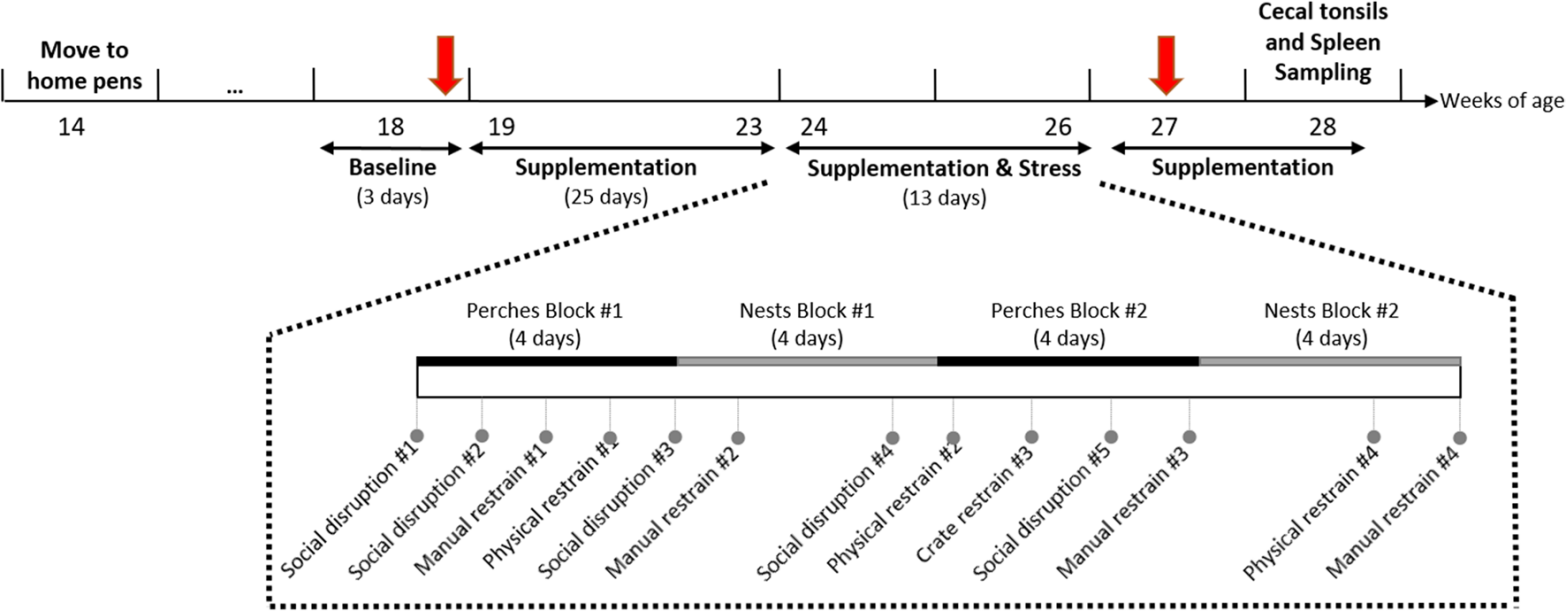
Schematic diagram of experimental timeline. Schematic diagram of experimental timeline. Laying hens were moved to the experimental pens at 14 weeks of age (woa). The supplementation with *L. rhamnosus* (Lacto) or a placebo (Placebo) started at week 19 and lasted until the end of the experiment. The stress treatment spanned weeks 24–26. Physical examinations, blood and fecal sampling (red arrows) were conducted at 18 woa (as a baseline) and 27 woa. Behaviors were recorded during week 18 and weeks 24–26. Spleen and cecal tonsils samplings were performed at 28 woa.

An overview of the experimental timeline is represented in Fig. 6. Half of the pens were systematically assigned to receive either an oral supplementation with 5 × 10^9^ *Lactobacillus rhamnosus* JB-1 (Lacto, n = 6 pens, 42 birds) resuspended in 1 mL of drinking water or a placebo of 1 mL drinking water (Placebo, n = 6 pens, 44 birds). *Lactobacillus rhamnosus* JB-1 was a gift from Alimentary Health Inc., Cork, Ireland to McMaster University. Between 19 to 26 woa birds were supplemented individually using 12 mL plastic syringes. Birds received treatments once a day, Monday to Friday mornings, between 9:00 and 10:30. To limit additional stress due to daily handlings, birds were trained, prior to the experiment, to enter a dog crate (93 × 61 × 58 cm) and receive supplementations using canned sweet whole kernel corn as a food reward.

Following 5 weeks of Lacto or Placebo supplementation, three pens from each group were randomly assigned to undergo a sequence of stressors (S, n = 6 pens, 42 birds) spanning a period of 3 weeks (Fig. 6). The remaining pens continued to receive Lacto or Placebo supplements in the absence of stress (NS, n = 6 pens, 44 birds). The five stressors used in this experiment were: social disruption (5 times), physical restraint of all individuals in a pen for 1 h in a transport crate (4 times), individual manual restraint for 5 min on a table outside of the experimental room (4 times), blocking nest boxes used for egg-laying for 4 days (twice), and blocking perches used for roosting for 4 days (twice). The 3-weeks stress regimen was designed to mimic the unpredictable and repeated stressors that hens encounter in commercial farm settings. These were reported as potential triggers for SFP in the literature^24,97–99^. The social disruption stressor consisted of splitting the pen into two subgroups of 3 to 4 individuals and mixing them with another subgroup from a different stressed pen that had received the same supplement (Lacto or Placebo). These newly mixed, socially disrupted, groups of birds were moved into new, identical pens with fresh bedding material.

### Behavioral observations and physical examination

Birds were individually identified using numbered silicone backpacks (8 × 6 × 0.5 cm) provided 2 weeks before the experiment started. Backpacks were fastened around the wings via two elastic straps secured to the backpacks with metal eyelets^100^. The time windows used to observe FP activity were determined by pilot observations. Baseline behavioral observations were recorded over three days during week 18 (5 min in the morning at 8:00 and 5 min in the afternoon at 17:00). During the simultaneous supplementation and stress treatments (weeks 24–26), birds were observed for 5 min at 8:00 and 10 min post-stressors. In total, each individual bird was observed for 185 min: 11 morning observations prior to supplementation treatment (5 min each) and 13 post-stress observations (10 min each).

All-occurrence sampling was used to record initiators and recipients of gentle and severe FP interactions. An occurrence was defined as a sequence of uninterrupted behavior lasting more than 4 s aimed at the same bird^60^. Gentle FP was defined as gentle peck(s) at the tips and edges of feathers of conspecifics without their removal. Severe FP was defined as intended, forceful peck(s) towards the feathers/body (not the head) of conspecifics that may remove feathers and cause injury^101^. All behavioral observations were performed by a trained, blinded observer.

Physical examinations to determine feather cover and bodyweight of each bird was performed during weeks 18 and 27 (Fig. 6). Feather cover was assessed using a severity scale from 0 (no or slight wear, nearly intact feathering) to 2 (at least one featherless area ≥ $2 Canadian coin) on the neck, back or tail^102^.

### Blood collection and analysis

Blood samples were collected (3.5 mL/hen) at 18 and 27 woa from the wing vein using EDTA-coated vacutainer tubes. Individual birds were sampled, one hour post-feeding, on the same day of the week and at the same time of day (between 10:00 and 14:00) during both sampling weeks. After collection, samples were stored on ice (maximum of 4 h) until centrifugation (4 °C, 2,500 rpm, 15 min) for plasma separation. Plasma was aliquoted into 1.5 mL microtubes and stored at − 80 °C until further analysis.

The concentration of amino acids and their derivatives, nitrite and neopterin were determined performed as reported previously^103,104^. Briefly, samples were analysed via reversed-phase HPLC using a LiChrosorb C18 column (5 μm particle size, Merck, Darmstadt, Germany) on a Varian ProStar system. For the separation of TRP and KYN, a 15 mmol/L acetic acid-sodium acetate solution (pH = 4.0) was used as the mobile phase. For sample preparation, protein was precipitated by adding 25 µl 2 mol/L trichloroacetic acid (Merck KGaA, Darmstadt, Germany) to 100 µl of sample. An aliquot of 100 µl of 3-nitro-L-tyrosine (25 µmol/L, Sigma Aldrich, Vienna, Austria) was also added as the internal standard. TRP was detected by its natural fluorescence at an excitation wavelength of 286 nm and an emission wavelength of 366 nm, KYN and 3-nitro-L-tyrosine were detected at a wavelength of 360 nm. To determine PHE and TYR concentrations, 30 μL of plasma was diluted with 30 μL of 0.015 mol/L potassium dihydrogen phosphate (Merck KGaA, Darmstadt, Germany), 300 μL of 500 μmol/L 3-nitro-L-tyrosine, and 75 μL of 2 mol/L trichloroacetic acid (for protein precipitation). After centrifugation, 370 μL of the supernatant was diluted with 400 μL potassium dihydrogen phosphate (0.015 mol/L), which was also used as the mobile phase. PHE and TYR concentrations were determined simultaneously by monitoring their natural fluorescence at an excitation wavelength of 210 nm and an emission wavelength of 302 nm.

Albumin-based mixtures of TRP, KYN, PHE and TYR (Sigma-Aldrich, Vienna, Austria) were prepared for external calibration using the same procedure used to analyze plasma samples. In mammals, the KYN:TRP ratio can be used to estimate TRP metabolism along the KYN axis. In humans, this ratio is used as an index of the IDO-1 enzyme mediated TRP breakdown when accompanied by an increase in markers (such as neopterin) of the cellular immune system^105^. PHE:TYR ratios were calculated as phenylalanine 4-hydroxylase (PAH) activity, which converts PHE to TYR^21^. TRP:(PHE + TYR) is a substitution for the commonly used ratio of TRP to the large neutral amino acids. As described in Wurtman et al.^106^, this ratio indicates the competition of TRP with other amino acids for uptake across the blood–brain-barrier. However, these findings relate to mammals and may not be fully translatable to poultry because of possible evolutionary variations^87,88^, and thus, the results should be approached with caution.

To estimate nitric oxide (NO) production, the stable NO metabolite nitrite was measured in the plasma sample collected using a modified Griess assay (Merck KGaA, Darmstadt, Germany). Neopterin levels were measured by enzyme-linked immunosorbent assay (ELISA) with a detection limit of 2.0 nmol/L (BRAHMS Diagnostics, Hennigdorf, Germany). Corticosterone concentrations were analysed using an ELISA kit (Enzo Life Sciences Inc., NY, USA). The intra- and inter-assay coefficients of variation based on controls were 1.5% and 6.5% for low quality controls, 0.9% and 2.5% for medium quality controls, and 0.9% and 1.4% for high quality controls, respectively.

### Sampling of cecal droppings and 16S rRNA sequencing

During weeks 18 and 27, birds were transferred to individual cages (45.7 L × 45.7 W × 40.5 H cm) for a maximum of 48 h. The hens had visual contact but no physical access to their neighbors. Clean foil trays were placed underneath each cage to collect droppings from each bird. After a minimum of 24 h, one gram of cecal feces material per bird was sampled using a clean spatula. Cecal excreta were identified by its characteristic homogeneous, smooth and creamy texture and dark colour. Samples were taken from the middle of the cecal discharge to avoid contamination with non-cecal excreta. The cecal samples were transferred into sterile 1.5 ml Eppendorf tubes and stored at – 80 °C until further processing of the sample.

DNA extraction was carried out as previously described^30,52^, using a modified protocol to increase recovery of bacteria across taxa^107^. 16S rRNA sequencing was carried out using a modified, barcoded Illumina sequencing method^108^. The 341F and 518R primers were used to amplify 16S rRNA from cecal excreta samples. A MiSeq Illumina sequencer in the McMaster Genome Center was used for paired-end reads of the V3 region^109^ and 250 nucleotide paired-end sequencing. An in-house bioinformatic pipeline was used to process the generated data^110^. Abundant Operational Taxonomic Units (OTUs) were used to produce clustered sequences of OTUs^111^, and taxonomic assignments were established using the RDP classifier and the GreenGenes training set^112,113^. Sequencing produced 5.960 OTUs, and a minimum, maximum, and median of 8,856, 126,407, and 56,768 reads/sample respectively. Data were rarefied at a sequencing depth of 8,856 reads/sample using QIIME^114^. The Shannon diversity index was calculated for alpha-diversity analysis, and Jackknife resampling was used to generate Bray–Curtis distances for beta-diversity analysis. (Dis)similarity between groups was calculated using Kruskal–Wallis H and Mann–Whitney U tests in GraphPad Prism 6.

### Spleen and cecal tonsil samplings and analysis

At week 28, 16 hens (4 Lacto-S; 4 Lacto-NS; 4 Placebo-S; 4 Placebo-NS, whereby S = stressed and NS = non-stressed) were killed by cervical dislocation. One cecal tonsil and the spleen were harvested from each bird within 3 min after death and kept in 5 mL of 5% Fetal Bovine Serum (FBS) containing RPMI medium. Spleen cells were isolated by scratching the spleen on a 40 µm cells strainer with a sterile syringe nozzle using sufficient pressure to completely crush the full spleen on a 6 well plate containing 6 mL cold Phosphate-buffered saline (PBS). Cell suspensions were then transferred into 15-mL falcon tubes and plates were rinsed with an additional 6 mL of cold PBS and pooled into the tubes. Cell suspensions were then centrifuged at 1,500 rpm at 4 °C for 10 min. Cells were then resuspended in red blood cell (RBC) lysis buffer for 3–4 min and washed with cold PBS at 1500 rpm for 10 min at 4 °C. Cells were resuspended in 5% FBS-RPMI medium. Cecal tonsils were cut into small pieces and digested in 2 mL of 5% FBS-RPMI containing 5 µM EDTA and 20 µg/mL Collagenase IV (Sigma Aldrich, Oakville, Canada) at 37 °C with shaking for 40 min. Then, cecal suspensions were passed through 40 µm strainer and washed with 5% FBS-RPMI medium by centrifuging at 1,500 rpm (4 °C) for 10 min. Cells were then resuspended in 5% FBS-RPMI medium. Viable spleen and cecal tonsil cells were counted by Trypan Blue exclusion and diluted in Fluorescence-activated cell sorting (FACS) buffer (PBS + 2% FBS) to a concentration of 10^6^ cells/ml. Both splenocytes and cecal tonsil cells were stained for regulatory T cells (Treg) markers using the following antibodies conjugated to their respective dyes: Mouse Anti-Chicken CD3—APC, Mouse Anti-Chicken CD4 -PE, Mouse Anti-Chicken CD8α-PerCP Cy5.5 (Southern Biotech, Birmingham, AL, USA) and Human Anti-Chicken CD25-FITC (BIO-RAD, Mississauga, ON Canada). Data were acquired using FACSCelesta (Becton Dickinson, Oakville, ON, Canada) and analysed using FlowJo (TreeStar, Ashland, OR, USA).

### Statistical analysis

FP frequencies were determined per individual per min and averaged at the pen level. Feather cover was scored across different body regions and these scores were combined to determine presence (at least one score > 0) or absence (all regions scored 0) of plumage damage.

The SAS software (version 9.4, SAS Institute, Cary NC) was used for all statistical computations except for the microbiota and immunological data, which were analyzed with GraphPad Prism 6 and SPSS using a two-tailed Student’s t-test, Mann–Whitney U-test, Kruskal–Wallis test, or ANOVAs, with Dunn’s multiple comparisons post-hoc tests. The assumptions of normally distributed residuals and homogeneity of variance were examined graphically with the use of QQ plots. Data was transformed where necessary. Statistical significance was considered at P < 0.05 and tendencies are reported when 0.05 ≤ P ≤ 0.1. Values are presented as least square (LS) means ± standard error, unless stated otherwise.

A generalized linear mixed model (PROC GLIMMIX) was used to assess the effect of supplementation (Lacto, Placebo) under stressed (S) versus non-stressed (NS) conditions. For each outcome, body weight and baseline values (i.e., behavior or physiological measures collected at 18 woa) were used as covariates. Differences between LS means were compared pairwise using a Tukey–Kramer adjustment. Scatter plots of studentized residuals against predicted values, and treatment values and a Shapiro–Wilk test of normality were used to confirm the assumptions of the variance analysis. To detect possible outliers, studentized residuals outside a ± 3.4 envelope were used. To identify whether blood measurements were interrelated with the severe FP phenotype, an additional GLIMMIX model was performed for each blood parameter with the phenotype as a fixed effect.

## Supplementary Information

Supplementary Information.

## Data Availability

All data necessary to evaluate the conclusions of this paper are available from the following online repositories: https://dataverse.scholarsportal.info/privateurl.xhtml?token=9050f262-d6bc-4bcd-8699-9121976dddc8.
