# Supplementary material for: Ingestion of Lactobacillus rhamnosus modulates chronic stress-induced feather pecking in chickens
Source: Sci Rep. 2021 Aug 24;11:17119. doi: 10.1038/s41598-021-96615-x (PMC8384842; doi:10.1038/s41598-021-96615-x)

**Ingestion of *Lactobacillus rhamnosus* Modulates Chronic Stress-Induced Feather Pecking in Chickens**

Claire Mindus, Nienke van Staaveren, Aadil Bharwani, Dietmar Fuchs, Johanna M. Gostner, Joergen B. Kjaer, Wolfgang Kunze, M. Firoz Mian, Anna K. Shoveller, Paul Forsythe and Alexandra Harlander-Matauschek

Supplementary Materials:

Supplementary Figure:

**Supplementary Figure S1.** Log2-fold change in the relative abundance of individual operational taxonomic units (OTUs) in cecal samples of 27-week-old stressed laying hens supplemented with Lacto (*L. rhamnosus*, n=40) relative to hens receiving the Placebo treatment (Placebo, n=44). Supplementation was carried from 19 weeks of age until cecal collection and the stress treatment spanned weeks 24-26. Values are indicated after adjusting the P-value for multiple comparisons (using False Discovery Rate technique), and after eliminating microbial groups that were already different between the two groups at baseline (week 18). Positively enriched OTUs are more abundant, on average, in the Lacto treated groups than in Placebo groups while negatively enriched OTUs are more abundant in the Placebo. OTUs are shown at the genus level or when unidentified at the genus level (UN) at the family level.


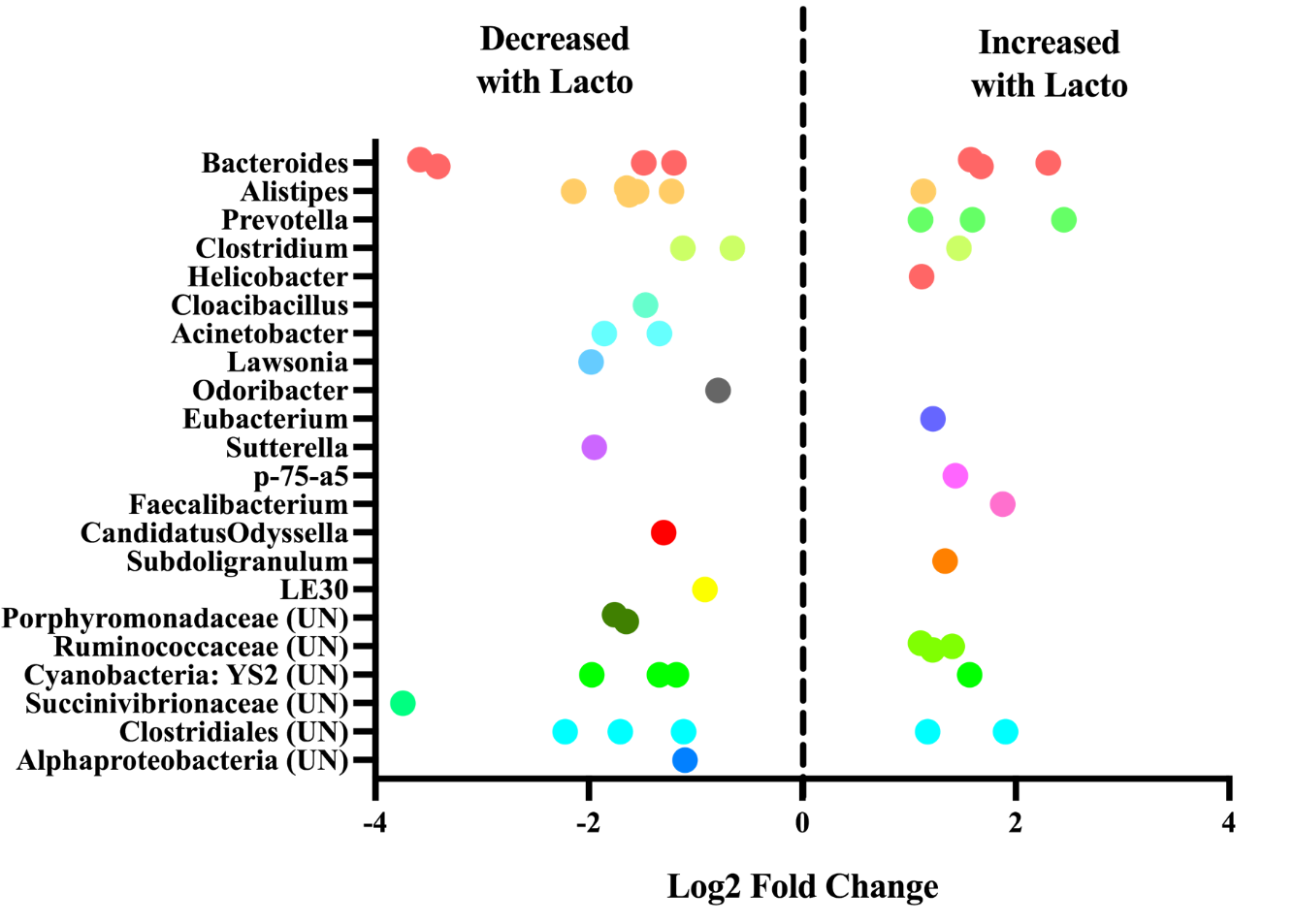


**Supplementary Figure S2.** Flow cytometry gating strategy to identify subsets of immune cells in the (a) spleen and (b) cecal tonsils of laying hens at 28 weeks of age. Lymphocytes were gated based on forward and side scatter. Lymphocytes were divided into CD3 positive T cells (CD3+). CD3+ T cells were further separated into CD3+CD4+, CD3+CD8+ and CD4+CD25+ T cells.


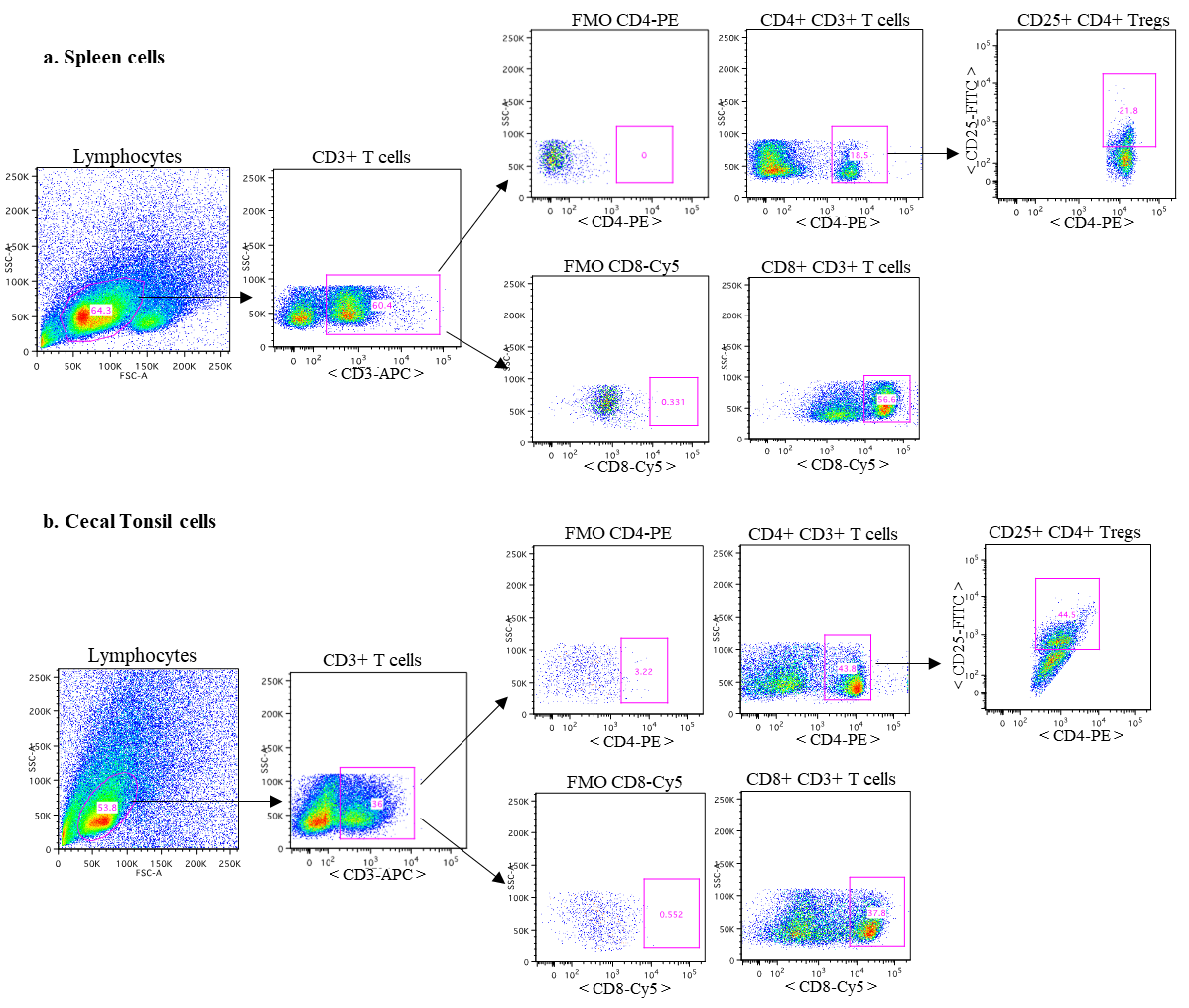

Supplement: Supplementary file 1 — Supplementary Information. [file 41598_2021_96615_MOESM1_ESM.docx]
